# The advantages of artificial intelligence-based gait assessment in detecting, predicting, and managing Parkinson’s disease

**DOI:** 10.3389/fnagi.2023.1191378

**Published:** 2023-07-12

**Authors:** Peng Wu, Biwei Cao, Zhendong Liang, Miao Wu

**Affiliations:** ^1^College of Acupuncture and Orthopedics, Hubei University of Chinese Medicine, Wuhan, Hubei, China; ^2^Hubei Provincial Hospital of Traditional Chinese Medicine, Wuhan, China; ^3^Affiliated Hospital of Hubei University of Chinese Medicine, Wuhan, Hubei, China; ^4^Hubei Academy of Traditional Chinese Medicine, Wuhan, Hubei, China

**Keywords:** Parkinson’s disease (PD), artificial intelligence, freezing of gait, motor evaluation, machine learning

## Abstract

**Background:**

Parkinson’s disease is a neurological disorder that can cause gait disturbance, leading to mobility issues and falls. Early diagnosis and prediction of freeze episodes are essential for mitigating symptoms and monitoring the disease.

**Objective:**

This review aims to evaluate the use of artificial intelligence (AI)-based gait evaluation in diagnosing and managing Parkinson’s disease, and to explore the potential benefits of this technology for clinical decision-making and treatment support.

**Methods:**

A thorough review of published literature was conducted to identify studies, articles, and research related to AI-based gait evaluation in Parkinson’s disease.

**Results:**

AI-based gait evaluation has shown promise in preventing freeze episodes, improving diagnosis, and increasing motor independence in patients with Parkinson’s disease. Its advantages include higher diagnostic accuracy, continuous monitoring, and personalized therapeutic interventions.

**Conclusion:**

AI-based gait evaluation systems hold great promise for managing Parkinson’s disease and improving patient outcomes. They offer the potential to transform clinical decision-making and inform personalized therapies, but further research is needed to determine their effectiveness and refine their use.

## Graphical Abstract 1

For the gait analysis based on AI technology, the data is collected first with the non-wearable or wearable devices. Afterward, the collected data is preprocessed, including sliding windows, denoising, normalization, etc. Subsequently, features are extracted with multiple methods. Finally, different classification algorithms are applied for the automatic recognition of FOG prediction, detection, and PD diagnosis and staging. Created with BioRender.com. 
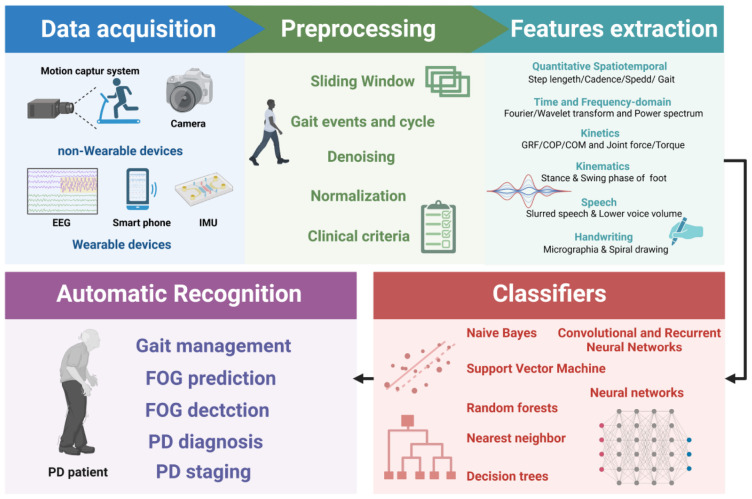

## Introduction

Parkinson’s disease (PD) is one of the most frequent neurodegenerative disorders that presents various life-altering symptoms, such as upper-limb trembling. The number of people affected by PD has doubled from 2.5 to 6.1 million between 1990 and 2016 (GBD 2016 Neurology Collaborators, 2019). The characteristics of advanced stages of PD (the Hoehn and Yahr Scale) are sever motor or non-motor complications, as well as limited mobility and decreased independence (Pedersen et al., 2017). The upper body postures of these patients are flexed, and their mass center is anteriorly shifted, with a small shuffle of steps, decrease of walking speed, and increase of gait variability (Hausdorff et al., 1998; Sofuwa et al., 2005; Baltadjieva et al., 2006; Hausdorff, 2009; Macht et al., 2010). As PD progresses, locomotion can deteriorate into freezing of gait (FOG), which is defined as the sensation that one’s feet are likely glued to the floor and unable to initiate the next step (Snijders et al., 2008; Macht et al., 2010). Notably, FOG can lead to poor balance and falls, obviously reducing quality of life (Adkin et al., 2003; Bloem et al., 2004; Young and Mark Williams, 2015). Therefore, early diagnosis and intervention of FOG is crucial for PD patients.

At present, clinical features serve as core basis of PD diagnosis (Loh et al., 2021). These clinical criteria mainly depend on the expertise of a neurologist, but are still not perfect with several limitations. For instance, the diagnostic accuracy of them has been reported to be slightly above 80% even in a specialist neurology center, compared to pathological examination (the gold diagnostic standard for PD) (Rizzo et al., 2016). Furthermore, a wide shortage of neurologists extends the waiting time for patients to get identified with PD, especially in countries with a large aging population and a high prevalence of neurological disorders (Burton, 2018). In addition, the dopaminergic neurons have been reportedly lost by 60% at the time of diagnosis (Balestrino and Schapira, 2020). Accordingly, early diagnosis is the goal of a large number of global research, to ensure optimal functional outcomes of PD patients (Loh et al., 2021).

In the last decade, PD pharmacological therapy has made great progress, yet without curative treatments (Bloem et al., 2021). Drugs may induce motor complications, including dyskinesia, and individuals may exhibit motor features of resistance for levodopa, such as treatment-resistant tremor, swallowing and speech disorders, and postural instability (Balestrino and Schapira, 2020). In order to overcome the shortcomings of single therapy, comprehensive guidelines consists of pharmacological treatment, non-pharmacological treatments, rehabilitation, and psychosocial supports have been widely applied for PD therapy. Among them, exercise management has been demonstrated to be able to improve both motor and non-motor features of PD and is particularly crucial for current and future treatment of PD (van der Kolk and King, 2013).

In recent years, artificial intelligence (AI)-based technologies, which have produced a marked effect in automated detection of seizures, atrial fibrillation, or computer-aided diagnosis, are emerging as one of the most promising way to ameliorate diagnosis and prognosis of PD (Tran et al., 2019). Besides, medical tools based on machine learning or deep learning can utilize PD biomarkers, particularly posture analysis in the gait cycle, to perform automated detection (Tuncer et al., 2019). Moreover, AI-based gait evaluation has emerged for predicting and preventing imminent FOG episodes, contributing to reduced fall-related injury and fear, and increased independence of PD patients (Nieuwboer, 2008; Ginis et al., 2018). Occurring FOG can be overcome by cueing devices based on AI that provide an external stimulus, including auditory, visual, and tactile; (Nieuwboer, 2008), for example, wearable sensors have been used to detect PD symptom to prevent freezes or reduce their effect using gait monitoring and assistive devices (Pardoel et al., 2019). Gait management referring to robotic systems is helpful for patients to maintain independent motor function and walk ability, improving the quality of life (Perju-Dumbrava et al., 2022).

Current studies have preliminarily reported the widespread use and effectiveness of gait evaluation AI-based systems in PD (Silva de Lima et al., 2017). However, automatic and reliable methods for clinical practice is far from resolved. A systematic search was conducted on PubMed and WOS for studies published before October 2022 using predefined keywords (“advanced PD,” “AI,” “machine learning,” “Freezing of Gait,” “deep learning” or “home-based telemedicine”) searching for studies published until October 2022. The purpose of this review is to present an up-to-date information and to elaborate the critical roles of AI-based gait evaluation in the early diagnosis, prediction, and management of PD (shown in Graphical abstract). Furthermore, details on the study populations, features used, and classification methods are also provided to promote clinical translation. By assessing the current state of the art and highlighting challenges and limitations, this review also identify knowledge gaps that need to be filled for future AI-based technologies, thus improving the quality of PD patients’ lives.

## Diagnosing PD with AI-based gait evaluation

As involuntary motor control is a major characteristic in PD, an assessment of gait can be utilized for PD diagnosis. In theory, gait is considered as the walking patterns of a person. In the case of PD, the disease progresses as the stiffness of the body and postural instability are increased, resulting in gait disturbance (di Biase et al., 2020). In this respect, deep learning models can be trained with the gait features and then used for the detection of PD. Kinetic and kinematics features are key features of gait. The former includes ground reaction force, and the latter includes stance and swing phase of the foot (Xia et al., 2020).

Methods for FOG detection vary in complexity, and a variety of models have been used in wearable sensor based on AI (Maachi et al., 2020). To better distinguish the FOG gait of a typical PD patient, some features have been attempted to improve classification performance, such as Fourier transforms, Moore et al. (2008, 2013); Delval et al. (2010); Zach et al. (2015); Capecci et al. (2016) freeze index, Moore et al. (2008) wavelet transforms, Handojoseno et al. (2015); Rezvanian and Lockhart (2016); Punin et al. (2019) freezing of gait detection on glasses, Ahn et al. (2017) and the widely used freezing of gait criterion; (Coste et al., 2014) also, multiple machine-learning techniques have been applied to improve detection performance, such as neural networks, Ahn et al. (2017); Camps et al. (2018) random forests, Tripoliti et al. (2013) nearest neighbor, Mazilu et al. (2012) decision trees, Mazilu et al. (2016); Camps et al. (2018) naïve Bayes, Tripoliti et al. (2013) and support vector machines (SVM) (Ahlrichs et al., 2016; Rodríguez-Martín et al., 2017a,b; Sama et al., 2017). Apart from that, unsupervised machine learning and anomaly detection have also been attempted without extensive exploration (Pham et al., 2017b).

Decision trees are composed of series of binary selections which can form branch structures resembling a tree. Currently, more complex decision trees, such as boosting techniques and ensembles of trees, have improved the performance in FOG and PD detection with the sensitivity ranging from 66.25 to 98.35% while specificity ranging from 66.00 to 99.72% (Tripoliti et al., 2013; Mazilu et al., 2016; Camps et al., 2018; Pardoel et al., 2019). SVM is a binary (two class) classifier which trace a plane and can separate data points from each class, then it can classify new data points based on their side of the plane. SVMs for FOG detection have been reported to perform results ranging from 74.7 to 99.73% for sensitivity and 79.0–100% for specificity (Ahlrichs et al., 2016; Rodríguez-Martín et al., 2017a,b; Sama et al., 2017; Pardoel et al., 2019). Since freezing and motor symptom manifest differently in PD for each person, for AI-based gait evaluation, person-specific models are always reported to outperform person-independent models (Mazilu et al., 2016; Rodríguez-Martín et al., 2017b). However, obtaining enough data to develop an individual model is difficult in clinical practice. Unsupervised learning may have the powerful ability to address this small dataset problem, as FOG episodes labeled by experts are not needed for these methods. Instead that, the classes are defined by clustering techniques, or the normal class is firstly defined using anomaly detection approach and then abnormalities that do not conform to that class, such as FOG, are identified (Mohammadian Rad et al., 2018). Although unsupervised FOG detection approaches are appealing, as data labeling is not required, Mohammadian Rad et al. (2018) have suggested that the performance of unsupervised models is worse than that of supervised models. More recently, transfer learning and semi-supervised learning have been proposed to establish partly personalized FOG detection approaches without large amounts of data (Torvi et al., 2018). The former uses a network that has been previously trained as a base to adapt the model to a new task, and the latter was trained using both labeled and unlabeled data. Torvi et al. (2018) used transfer learning to train a neural network based on group data, and then they added an additional network layer trained with an individual’s data. By this way, the automatic and personalized detection has been realized. Some studies have reported the performance of semi-supervised learning applied to FOG detection are ranged from 89.2 to 95.9% in the sensitivity and 93.1 to 95.6% in the specificity (Mikos et al., 2017; Pardoel et al., 2019). Advantages of both supervised and unsupervised learning are combined in semi-supervised learning, indicating that semi-supervised learning allows person-specific tuning while preserving the generalization ability from a multiple person data set. Nevertheless, the value of semi-supervised learning for FOG detection in patients with PD remains unclear, more studies are needed to clarify the clinical potential of it for FOG detection in the future.

Handwriting and speech are also movement-related tests in PD detection. Micrographia refers to the phenomenon where the typefaces is smaller than normal due to a reduction in handwriting, which is a possible symptom in most PD patients (McLennan et al., 1972). The spiral drawing test is the most common test used in the literature for PD detection based AI (Drotár et al., 2016; Pereira et al., 2016). Kamran et al. (2021) have tested and fine-tuned the transfer learning architectures of CNN (convolutional neural networks) to differentiate handwritten drawings of PD from normal individuals, with the model accuracy of 99.22%. Besides, PD patients may exhibit slurred speech and lower voice volume (Tjaden, 2008). In two recent studies, using voice aberration has achieved higher than 99% accuracy to diagnose PD based on AI technique (Nagasubramanian and Sankayya, 2020; Goyal et al., 2021). Moreover, Ali et al. (2019) have proposed a genetically optimized neural network termed as LDA-NN-GA comprising linear discriminant analysis (LDA) for dimensionality reduction and genetic algorithm (GA) for hyperparameters optimization of neural network (NN) which is used as a predictive model. By using all the extracted features from the dataset collected by Sakar et al. (2013), the novel LDA-NN-GA achieved 95% classification accuracy on training database and 100% on testing database. Furthermore, in order to obtain unbiased results, the gender dependent features were eliminated, and the accuracy for training and testing database were 80 and 82.14%, respectively, indicating that LDA-NN-GA could well classify PD patients from healthy individuals. The above findings indicate that AI-based handwriting and speech recognition has great diagnostic accuracy for PD patients, however, more clinical studies with large populations are urgent before its clinical application.

Considering that single modality has certain limitations, multi-model analysis of PD, not limited to single type of modality, may be a useful tool for neurologists. By combing three input signals, including gait, handwriting, and speech, Vasquez-Correa et al. (2019) reported a CNN model with the accuracy of 97.6% for the diagnosis of patients with PD. In addition, Oung et al. (2017) have used both motion and speech data as input signals for PD early detection, and proposed an extreme learning machine with only one hidden layer in its network, which reached 95.9% classification accuracy and comparable to that obtained by Vasquez-Correa et al. (2019). Moreover, by introducing appropriate features deletion or addition, the performance of this proposed methodology might be strengthened in the furutre. Notably, the extreme learning machine can randomly select the most optimal hidden neurons and only needs a single iteration for model training, leading to a faster training and a less overfitting problem (Ding et al., 2015). Accordingly, to develop methods dependent on multiple input signals, instead of single modality for PD detection should be the focus of future studies, thus shedding light on the clinical transformation of multi-model analysis for PD patients. Table 1 has summarized the essential features of the above related studies.

**TABLE 1:** Diagnosing PD with AI-based gait evaluation.

Sample size	Objective	Data acquisition	Machine learning algorithms	Outcome variables	References
21 PD patients	Detecting FOG episodes in PD patients	A single IMU with three tri-axial sensors: accelerometer, gyroscope and magnetometer	Tree bagging, AdaBoost, LogitBoost, RUSBoost, RobustBoost and SVM	The deep learning model achieved 90% for the geometric mean between sensitivity and specificity	Camps et al., 2018
16 (5 healthy, 5 PD patients with FOG, and 6 PD patients without FOG	To detect FOG events in patients suffering from PD	Wearable sensors (six accelerometers and two gyroscopes)	Naïve Bayes, Random Forests, Decision Trees and Random Tree	81.94% sensitivity, 98.74% specificity, and 96.11% accuracy	Tripoliti et al., 2013
18 PD patients with a history of FOG	Investigating whether wrist motions during walking are correlated with FOG, and whether wrist-attached wearable sensors can be used to detect FoG episodes	Wrist mounted IMUs	C4.5 classification models	FOG can be detected by using wrist motion and machine learning models with a FOG hit rate of 0.9, and a specificity between 0.66 and 0.8	Mazilu et al., 2016
20 PD patients (8 patients presented FOG episodes and 12 did not)	To monitor FOG episodes based only on acceleration measurements obtained from a waist-worn device	Waist-mounted sensor	SVM for training or classification	Using a linear SVM kernel provides 98.7% accuracy and a geometric mean of 96.1%	Ahlrichs et al., 2016
21 PD patients	To develop a machine learning approach to monitor FOG during the daily life of PD patients	A single tri-axial accelerometer worn at the waist.	SVM	Enhancement in the specificity and sensitivity GM of 7.2%.	Rodríguez-Martín et al., 2017b
25 PD patients	Designing an IMU, called 9 × 3 to assess PD symptoms at homes during long periods	A single waist mounted system	A leave-one-patient-out with an SVM based on a radial basis function kernel	Exhibiting a sensitivity and specificity over 80%	Rodríguez-Martín et al., 2017a
15 PD patients	Presenting a new methodology to detect FOG	A single inertial system located at the waist	SVM and Logistic regression	91.7% of sensitivity and 87.4% of specificity, enhancing the results of former methods between a 5% and 11% and providing a more balanced rate of true positives and true negatives	Sama et al., 2017
/	To study domain adaptation algorithms to predict FOG episodes in patients with PD	Daphnet freezing of gait dataset	LSTMs, RNNs	The LSTM networks outperformed RNNs, with accuracy around 80%	Torvi et al., 2018
/	Presenting a method for early diagnosis of PD using patients handwriting samples.	Parkinson’s Handwriting Datasets, including PaHaW, HandPD, NewHandPD and Parkinson’s Drawings dataset	Transfer learning architectures of CNN	Achieving PD identification performance with 99.22% accuracy	Kamran et al., 2021
/	Using acoustic-based DL techniques to detect PD symptoms	Parkinson telemonitoring dataset and multi-variate sound record dataset	DMVDA System combining ADNN, ADRNN, and ADCNN	DMVDA produced 3 to 5% increase than the existing techniques	Nagasubramanian and Sankayya, 2020
/	Proposing a hybrid approach to extract features from resonance-based and time-frequency based information	Public Dataset (D1) “Mobile Device Voice Recordings at King’s College London (MDVR-KCL) from both early and advanced Parkinson’s disease patients and healthy controls” dataset, and dataset (D2) collected from 20 HC in India to show the effect of diversity on the diagnosis process.	Combination of Resonance based Sparse Signal Decomposition (RSSD) + Time-Frequency (T-F) algorithm CNN	Distinguishing PD and healthy with a validation accuracy of 99.37%	Goyal et al., 2021
/	Developing a hybrid intelligent system to automatically analyze voice signals to detect PD	The dataset used in this study was collected by Sakar et al. (2013)	LDA-NN-GA composed of LDA, GA and NN	Classifying PD patients from healthy individuals with 82.14% accuracy in testing database under unbiased condition	Ali et al., 2019
44 PD patients and 40 healthy subjects	Establishing multimodal assessment of PD	Recordings of speech, handwriting, and gait collected from the study samples	Architectures based on CNNs	The fusion of the three bio-signals achieved accuracy of 97.6%	Vasquez-Correa et al., 2019
65 subjects (15 healthy subjects, 50 PD patients)	To detect and classify PD using signals from wearable motion and audio sensors	Wearable IMU known as Motion Node Bus and a headset (Sennheiser DW Pro2)	KNN, PNN and ELM	Exhibiting over 90% accuracy to differentiate PD from non-PD subjects	Oung et al., 2017

FOG, freezing of gait; IMU, Inertial Measurement Unit; SVM, support vector machine; CNN, convolutional neural network; RNN, recurrent neural network; LSTM, long short-term memory; KNN, K-nearest neighbors; PNN, probabilistic neural network; ELM, extreme learning machine.

## Predicting FOG with AI-based gait evaluation

Freezing of gait is frequently encountered in Parkinsonian disorders, certain studies in recent years have thus made a great attempt to predict its occurrence and development. These studies varied in approach and performance and focused more on understanding the complexity of FOG prediction. In addition to factors that are considered in FOG detection studies, such as dataset size, FOG definitions, medication state, and contextual or study-specific performance metric definitions, data before freeze onset must be collected to define the pre-FOG class in FOG prediction studies. In other words, the data from the pre-FOG class is recognized by a machine-learning model to typically predict FOG. However, the subtle transition from walking to FOG makes it difficult to label the start of pre-FOG, which is a single fixed duration predetermined to select data prior to the FOG episode and identified from visual observation. Generally, a pre-FOG segment duration of 1–6 s has been widely accepted in FOG prediction studies (Handojoseno et al., 2015; Pardoel et al., 2019). For example, Handojoseno et al. (2015) used a 5 s period and Palmerini et al. (2017) applied a 2 s period as the pre-FOG segment. Notably, distinguished from other studies, Mazilu et al. (2015) used an assumed 3 s period for pre-FOG feature selection and created a person-specific, anomaly detection model based on multivariate Gaussian distribution, which could be manually tuned for each participant.

Despite that, the optimal pre-FOG segment duration is still not clear at present. There exists a hypothesis that the pre-FOG segment is a linear degradation of gait resulting in FOG (Heremans et al., 2013). According to this threshold theory, data closest to the FOG would resemble freeze, while data farther from the FOG would represent typical PD walking. For this reason, when using a two-class classifier to discriminate pre-FOG from typical PD walking, pre-FOG segment should be short (Torvi et al., 2018). However, Mazilu et al. (2013b) proposed that for a three-class classifier composed of FOG, pre-FOG, and typical PD walking classes, a short segment before FOG might not be ideal, because it was much difficult to distinguish between the pre-FOG and FOG classes if pre-FOG segments is very short. Although a longer pre-FOG segment can improve the classification of pre-FOG, the accuracy of FOG and typical walking classification is greatly reduced. Hence, how to determine the pre-FOG period in a three-class classifier is urgent to balance the classification accuracy among pre-FOG, FOG and typical walking. Across different participants or different FOG episodes for the same individual, the optimal pre-FOG duration varies with best performance (Mazilu et al., 2013b). In their observational studies, Mazilu et al. (2015) and Palmerini et al. (2017) also supported that a single pre-FOG segment duration is inadequate. Therefore, pre-FOG detection and prediction performance can be improved by a person- or episode-specific pre-FOG duration. This will be beneficial for reducing the overlap with walking class and elevating the purity of class containing only pre-FOG data.

In PD-related studies, neural networks have been frequently used for FOG prediction based on gait evaluation. They are inspired by neuron structure in the brain and are composed of interconnected layers of nodes (Parisi et al., 2019). Compared with neural networks for FOG detection, prediction performance tended to slightly worse. Recently, the performance of neural networks model has been reported to achieve 72.2–99.8% sensitivity and 48.4–99.9% specificity for FOG detection, with sensitivity of up to 86%, specificity of 80.3%, and precision of 89% for FOG prediction (Handojoseno et al., 2015; Saad et al., 2017; Camps et al., 2018; Pardoel et al., 2019). CNN, recurrent neural networks (RNN), and other different subtypes of neural networks have also been widely used in FOG prediction (Camps et al., 2018; Mohammadian Rad et al., 2018; Torvi et al., 2018). CNN can identify local patterns within images but without the requirement of features selection prior to implementation (Anwar et al., 2018; Yamashita et al., 2018). They have become popular in numerous FOG detection studies (Shalin et al., 2020; Filtjens et al., 2021). For instance, a deep learning based on CNN for FOG detection in PD patients by using a waist-worn IMU exhibited 91.9% sensitivity and 89.5% specificity (Camps et al., 2018). RNN utilizes both current inputs and previous data during classification, thus giving the network with the ability of “memory” to help recognize sequences (Sak et al., 2014; Anwar et al., 2018). Since RNN is suitable for time-series data, it has been employed for the prediction of FOG, especially in recent years. Torvi et al. (2018) have applied a special type of RNN, long short-term memory (LSTM) (Hochreiter and Schmidhuber, 1997), to predict FOG, with a reported accuracy of more than 90% for predicting FOG 5 s in advance. In recent years, several novel and efficient algorithms have been continuously attempted. Arami et al. (2019) proposed a binary classification by a feature time series prediction and suggested it as a standard, since a significant improvement has been demonstrated when comparing with the conventional three-class prediction model. Another useful approach, involving plantar pressure data in a 2D-image form and evaluated by CNN, has been proved accurate to predict the aforementioned incident (Shalin et al., 2020).

The continued development of FOG prediction systems plays a critical role in long-term monitoring and real-time cueing, as well as in implementation in gait-assist systems. While high-performing methods have been steadily increasing for FOG prediction, there still some great challenges. The representation of inconsistent nature of FOG will benefit from a set of diverse characteristics in both the time and frequency domains. Besides, supplying an element of personalization upon the established prediction methods for FOG may be a promising approach for future research. Furthermore, the sample size of the gait database is relatively small, and there is an imbalance of gender, age and stages in the dataset. Notably, the data is always collected with the patient’s knowledge, which may cause unconscious posture changes and affect the accuracy of the final data. Therefore, how to obtain gait information of subjects in their natural state is very essential for improving the quality of database. Table 2 illustrated these research for predicting FOG with AI-based gait evaluation.

**TABLE 2:** Predicting FOG with AI-based gait evaluation.

Sample size	Objective	Data acquisition	Feature extraction	Pre-FOG segment	Outcome variables	References
16 PD patients with a FOG history	Evaluating the potential of Electroencephalography (EEG) Brain Dynamics in analyzing and predicting FOG	The EEG was recorded using a 4-channel wireless EEG system with gold cup electrodes	EEG Linear Univariate Measurements, EEG Non-Linear Univariate Measurements, and EEG Bivariate Measurements	5 s	This combination resulted in a sensitivity of 86.0%, specificity of 74.4%, and accuracy of 80.2% when predicting episodes of freezing, outperforming current accelerometry-based tools for the prediction of FOG	Handojoseno et al., 2015
11 PD patients with a FOG history	Presenting a new approach for the prediction of FOG (before it actually happens)	Wearable inertial sensors, specifically accelerometers and gyroscopes	Computed from the signals recorded by the inertial sensors	2 s	Demonstrating a degradation of gait occurring before freezing, and providing preliminary evidence on the feasibility of creating an automatic algorithm to predict FOG	Palmerini et al., 2017
18 PD patients	Developing an anomaly based algorithm for predicting gait freeze from relevant skin conductance (SC) features	CuPiD multimodal dataset and Actiwave1 (for ECG collection)	Features were extracted in a sliding- window manner	3 s	Predicting 71.3% from 184 FOG with an average of 4.2 s before a freeze episode happened	Mazilu et al., 2015
/	To develop feature learning for detection and prediction of FOG in PD	DAPHNet dataset	Supervised Domain- specific Feature Extraction, Supervised Feature Extraction of Time-domain and statistical features and unsupervised Feature Learning	1–6 s	For different participants or different FOG episodes for the same individual, the optimal pre-FOG duration varies with best performance	Mazilu et al., 2013b
21 PD patients who manifested FOG episodes	To develop a DL for FOG detection in PD patients	The inertial data were recorded using a single IMU with three tri-axial sensors: accelerometer, gyroscope and magnetometer	MBFA, Online FOG detection, Four-stage FOG detection and FOG detection for home environments	/	The DL based on CNN for FOG detection in PD patients exhibited 91.9% sensitivity and 89.5% specificity	Camps et al., 2018
/	To study the performance of advanced DL algorithms to predict FOG events in short time durations before their occurrence	Daphnet Freezing of Gait dataset	LSTM (RNN)	1, 3, 5 s	More than 90% for predicting FOG 5 s in advance	Torvi et al., 2018
/	Presenting a novel technique to predict FOG in advance-stage PD using movement data from wearable sensors	Daphnet dataset	A set of time domain and frequency domain features were extracted from the 3D acceleration data	75 different time- and frequency-domain features were extracted from the raw accelerations. Features were extracted per sensor or per axis	A sensitivity of 93 ± 4%, specificity of 91 ± 6%, with an expected prediction horizon of 1.72 s	Arami et al., 2019
5 PD patients with a FOG history	To develop a novel method of FOG prediction with plantar pressure data treated as 2D images and classified using a CNN	Participants walked a predefined freeze-provoking path up to 30 times for data collection	CNN. MATLAB R2019b	0.5, 1, 1.5, 2, 2.5, 3 s	The model detected FOG before the event, with good results at 0.5, 1.0, and 1.5 s intervals	Shalin et al., 2020

FOG, freezing of gait; CNN, convolutional neural network; DL, deep learning; MBFA, Moore-Bächlin FOG algorithm; LSTM, long short-term memory.

## AI-based gait management in PD patients

Maintaining independent motor function and walking capability is a primary goal of therapy for moderate to advanced PD patients. Through this personalized approach, patients are expected to keep independent for as long as possible, delay entering a worse condition where they are immobilized, and improve their quality of life. Specifically, muscle strength can be enhanced by resistance training, and the gait performance will be improved; (Picelli et al., 2012) shortening of flexor muscles can be reduced by stretching, thus the abnormally flexed posture will be alleviated in PD; postural control can be improved by balance exercise (alone or together with other training modalities), thus decreasing the falls risk. As a rhythmic motor activity, gait management is regarded as a specific intervention in physical therapy trials for PD. Previous studies have shown that improvements in gait measures, such as step length, cadence, etc., are helpful for postural control and falls risk reduction (Tomlinson et al., 2013, 2014; Mehrholz et al., 2015). These enjoyable methods offer the advantage to favor social engagement for PD patients.

In fact, physical intervention, including gait management, could not only help motor complications of PD but also facilitate neuroplasticity and behavior. Studies on animal models of PD have revealed the mechanism of a dynamic interplay between degeneration and regeneration, which could be induced by exercise and learning (Hirsch and Farley, 2009); dopaminergic and glutamatergic neurotransmission may be linked to activity-dependent processes, thus modulating cortically driven hyper-excitability. Fisher et al. (2013) recorded neuroplasticity of dopaminergic signaling in 4 individuals with early stage PD when they were practicing a treadmill exercise, and found that exercise-induced an increase in the dopamine D2 receptor (DA-D2R), as well as improved postural control in PD patients. The above findings suggested that exercise is responsible for the neuroplasticity in dopaminergic signaling and the improved postural control function in early stage PD. In mild to moderate PD subjects, learning-dependent gray matter changes in balance training are correlated with performance improvements shown in voxel-based morphometry, while the changes induced by forced exercise were comparable to medication (Beall et al., 2013; Sehm et al., 2014). Moreover, growing evidence has uncovered that by physical exercise, neurotransmitters and trophic factors synthesis are stimulated, and chronic oxidative stress are relieved as mitochondria biogenesis are increased and autophagy are enhanced; these neurochemical phenomena are closely associated with the improvement of neuroplasticity (Monteiro-Junior et al., 2015). Therefore, exercise management and training may induce brain plasticity, increased synaptic strength and potentiated functional circuitry, to improve behavior of PD patients (Petzinger et al., 2013).

In the past decade, AI-based applications for gait management and high-performing robotic systems have emerged exclusively for advanced PD. A pilot study revealed that robot-assisted gait training is a prospective method to against FOG and improve gait of PD patients (Lo et al., 2010). Nevertheless, more evaluations of the long-term effects with a further follow-up are recommended. Nardo et al. (2014) performed the same therapy sessions as in the aforementioned study on patients who have underwent deep brain stimulation (DBS) previously, and suggested that robot-assisted gait training might be constructive only for space-temporal gait parameters and motor score, but not for kinetic and kinematic gait parameters. Although PD patients may benefit from a robotic-assisted treadmill training, it is still need further investigation to compare their effects with conventional treadmill training. Encouragingly, trials on comparison between conventional and robot-assisted gait training have been started. Picelli et al. (2012) compared robotic stepper training with physiotherapy with active joint mobilization, and found a more positive effect of the robotic training. This statistically significant difference lasted for at least 1 month in favor of the robotic system and indicated the need for future comparison between robot-assisted gait training and the same amount of treadmill training or overground walking. The first study aimed at quantitatively comparing the effects of robot-assisted gait training (RAGT) in PD and treadmill training was conducted by Galli et al. (2016), in which gait kinematics and spatiotemporal parameters were evaluated. The intensive treadmill therapy group (IG) showed no obvious changes in either gait profile score (GPS) or gait variable scores (GVSs), whereas improved gait kinematics were identified, especially in the frontal plane at the pelvic and hip joint level in subjects undergoing robotic training. However, to better describe the effects of this training approach, more studies with a larger number of PD patients, and the assessment of gait kinematics are needed. Later, Kang et al. (2019) designed a comparative clinical trial to specifically assess the effects of robot-assisted gait training (Walkbot-S™). Positive roles of AI-based applications in gait speed and automaticity were revealed, as well as in balance function, fall risk, disease severity, and quality of life. In addition, by monitoring gait automaticity changes and brain functional network fluctuations, new light was shed on possible pathways through which these effects occur (Kang et al., 2019). Nevertheless, this study only carried out in one center which might not be consistent with other centers, thus various centers should be included to obtain more general results.

As for self-selected speed gait training, differences between robot-assisted gait training (Lokomat plus VR) and conventional gait training overground were not significant in a previous study (Fundarò et al., 2019). However, Capecci et al. (2019) took walking speed, endurance, number of FOG episodes, and general attitude toward the disease into consideration to compare robot-assisted gait training with treadmill training, and found that the frequency of daily FOG episodes was significantly decreased in the robot-assisted group, suggesting the great advantages of robot-assisted gait training in improving gait and endurance for PD patients. For a customized use of AI-based applications and robotic systems, as well as to add benefits to this treatment option, adjustment for body weight support, guidance force, and other parameter settings are warranted for future investigations.

Owing to prolonged exercise has been proven beneficial for motor learning retention, medium to long-term follow-up studies are valuable. Bevilacqua et al. (2020) enrolled 195 subjects, to explore the gait and balance improvement in older PD patients at 2 years following a 5-week rehabilitation. Briefly, all patients were divided into three groups in which 50-min traditional therapy programs were followed in the control group. The Tymo system was used in one technological intervention group, and the Walker View was applied in another intervention group with a traditional rehabilitation session for 30 min together with a robot-assisted treatment for 20 min. Finally, the evaluation of step length and asymmetry, walking and functional status, as well as acceptance of the technology will be employed for the efficacy of this novel treatment. This clinical trial focused on the utilization of robotic device, and examined the results both at the end and in the long term of the treatment, providing an innovative approach for the rehabilitation of patients with PD.

Another use of movement assessment devices may lie in differentiating distinct states of pharmacological treatment, such as under- or over-treatment of levodopa, coupled with gene expression data from patients diagnosed with PD (Turner et al., 2016). By these systems, key dysregulated modules could be identified and drugs that help for restoring homeostasis would be suggested (Yue et al., 2017). Other sensor-based devices may identify non-adherence to drug treatment and aid in decreasing its prevalence (Tucker et al., 2015). Consequently, AI-based gait evaluation has great potential in automatic management of PD patients with significant advantages on improving quality of care and reducing the cost of patients as well as healthcare systems (Table 3).

**TABLE 3:** AI-based gait management in PD patients.

Sample size	Objective	Training system	Intervention	Analysis	Outcome variables	References
5 patients with idiopathic PD	To examine the potential effect of continuous physical cueing using RAGT on reducing FOG episodes and improving gait.	Lokomat (a robot-assisted gait training system)	Participants received 10 sessions of robot-assisted body weight-supported treadmill training (BWSTT) on the Lokomat	With FOG-Q, FOG and falls diary, posture and gait score, gait parameters, CV, GA, PCI, PDQ-39 and vFOG	Reduction in FOG, and improvements in gait velocity, stride length, rhythmicity, and coordination	Lo et al., 2010
9 PD patients treated with DBS	Assessing the effects of a Robotic-Assisted Rehabilitation Protocol (RARP) on gait patterns	Lokomat (a robot-assisted gait training system)	All patients had 45 min session of Lokomat rehabilitation every day for 5 weeks	Gait assessment was performed 1 day before the first training session (PRE-RARP), and 1 day after the end of the rehabilitation protocol (POST-RARP) using an optoelectronic system with passive markers	Significant improvements on spatio-temporal gait parameters, but not kinematic and kinetic gait parameters	Nardo et al., 2014
41 PD patients	To compare the walking improved effect between RAGT and conventional physiotherapy	Robotic stepper training device (RST) group and physiotherapy group	A training program consisting of twelve 45-min sessions (including rest periods), 3 days, a week (Monday, Wednesday, and Friday) for 4 consecutive weeks	Primary evaluation: 10MWT and the 6MWT. secondary evaluation: The GAITRite system was used to evaluate spatio temporal gait parameters	RST group showed improved walking speed and distance	Picelli et al., 2012
55 PD patients	To compare the effects of end-effector robotic rehabilitation locomotor training versus intensive training with a treadmill	G-EO robot system, and intensive treadmill therapy	A cycle of outpatient rehabilitation treatment, consisting of at least one daily 3-h cycle	Clinical examination and 3D quantitative gait analysis at the beginning (T0) and at the end (T1) of the treatment	The end-effector robotic rehabilitation locomotor training improved gait kinematics and seems to be effective for rehabilitation in patients with mild PD	Galli et al., 2016
44 PD patients	Investigating the effects of RAGT on gait velocity and the underlying mechanisms	Walkbot-S robot system and treadmill training	RAGT group will receive gait training with the Walkbot-S for 12 sessions and treadmill training group receive gait training on the treadmill for 12 sessions	10MWT for primary outcome; the dual-task interference, BBS, TUG test, KFES-I, NFOG-Q, and MDS-UPDRS for secondary outcomes	This trial will compare the effects of RAGT with those of treadmill training on gait performance in patients with PD	Kang et al., 2019
96 PD patients	To compare the effects of RAGT and treadmill training on endurance and gait capacity in PD patients	The end-effector robotic device G-EO system	RAGT and treadmill training participant completed 20 sessions (5 days/week for 4 weeks)	6MWT, Timed Up and Go test, Freezing of Gait Questionnaire, Unified Parkinson’s disease Rating Scales and Parkinson’s Disease Quality of Life Questionnaire-39 administered before (T0) and after treatment (T1)	With RAGT, endurance and gait capacity were enhanced by 18% and 12%, respectively, and motor symptoms and quality of life were improved by 17% and 15%	Capecci et al., 2019
195 PD patients	Evaluating the effectiveness of robotic-based intervention of the older adults with PD	Tymo system or Walker View robot system and a traditional rehabilitation program	The control group performs traditional therapy sessions lasting 50 min. The Tymo system or Walker View group underwent 30 min of traditional therapy and 20 min with a robotic system	MMSE, RA, SHY, BI, FAC, SF-12, POMA, FES-I, PIADS, ATDPA, GDS, CDR and gait analysis and instrumental postural analysis	Proposing a new approach in the PD rehabilitation	Bevilacqua et al., 2020

RAGT, robot-assisted gait training; CV, GA, PCI, Gait Rhythmicity, Asymmetry, and Coordination; PDQ39, Parkinson’s disease questionnaire-39; vFOG, visual FOG; 10MWT, 10-minute walk test; 6MWT, 6-minute walk test; BBS, Berg Balance Scale; TUG, timed up and go; KFES-I, Korean version of the falls efficacy scale-international; NFOG-Q, new freezing of gait questionnaire; MDS-UPDRS, movement disorder society-sponsored revision of the unified Parkinson’s disease rating scale; MMSE, mini-mental state examination; RA, Rankin Scale; SHY, Hoehn and Yahr scale; BI, Barthel Index; FAC, functional ambulation categories; SF-12, SF-12 health survey; POMA, performance-oriented mobility assessment; FES-I, falls efficacy scale – international; PIADS, psychosocial impact of assistive devices scale; ATDPA, assistive device predisposition assessment; GDS, geriatric depression scale 5-items version; CDR, clinical dementia rating scale.

## Features used in AI-based gait evaluation in PD

Parkinson’s disease applications have used a variety of features in detection, prediction, and management, while the majority were previously proposed in non-PD applications (Caby et al., 2011; Shany et al., 2012; Howcroft et al., 2016, 2017). In general, K-index, Lorenzi et al. (2015, 2016); Kita et al. (2017); Suppa et al. (2017) R-index, Mazzetta et al. (2019) freeze index, Moore et al. (2008) K freeze index, Pham et al. (2017a) multichannel freeze index, Pham et al. (2017a) freezing of gait detection on glasses, Ahn et al. (2017) and the widely used freezing of gait criterion (Coste et al., 2014) are all custom features created for PD. Maximum acceleration amplitude within a window and rotation about a single axis are time domain features that are simply fast to compute (Palmerini et al., 2017). Gait-based features, including step length, cadence, and stride duration, are calculated from time domain data, Delval et al. (2010); Djuriæ-Jovici et al. (2014) as well as statistical features such as mean, standard deviation, and root mean square (Mazilu et al., 2016; Rodríguez-Martín et al., 2017a,b; Saad et al., 2017; Sama et al., 2017). Frequency domain features include peak amplitude and corresponding frequency, Ahlrichs et al. (2016) spectral density center of mass, Handojoseno et al. (2015); Sama et al. (2017) standard deviation in frequency domain, Rodríguez-Martín et al. (2017b) power of the signal in specific frequency bands, Bachlin et al. (2010) and freeze index were the most commonly used (Moore et al., 2008). Due to the limitations of Fourier transform, wavelet approaches are used more in recent years which are typically used for signal conversion from time domains to frequency domains (Rezvanian and Lockhart, 2016).

A wide range of gait manifestations can be better represented by a feature set, as better performance has been observed in studies combining time and frequency domains features compared with either individual type of feature (Handojoseno et al., 2015). Time domain features account for cadence, asymmetry, step length, peak limb angular velocity, and other gait parameters; (Delval et al., 2010) more subtle patterns of FOG, including trembling in specific frequency bands, however, are extracted by frequency domain features (Moore et al., 2008). The combined use of multiple features can always achieve the best performance.

It is of great importance to choose appropriate features for a real-time system where both classification performance and classification speed are required. For example, detection of a FOG episode could be delayed due to the calculated stride duration of approximately 1 s at the end of the stride. This limitation may also exist in other features, including cadence, step length, stride peaks, cadence variation, and freezing of gait criterion, depending on the method of feature calculation. Capturing features from windowed data with an appropriate size do not have this problem, since the calculation can be quickly performed at the time the data window is available. Calculation delay together with the step size of the sliding window determine the availability of features to the classifier. If processing power is sufficient, real-time applications could utilize almost all window-based features. However, given a limited computing power, complex features requiring excessive calculation stages may lead to unacceptable delays, which always occurs in multiple wearable systems. Although it is desirable to use a minimal quantity of easily calculated features, classification performance may become worse if features are too few or too simple. To balance classification performance and running speed, implementing feature selection algorithms is suggested for determination of the optimal features extracted from a larger set (Mazilu et al., 2015; Palmerini et al., 2017). Relief-F and related approaches have been reported to be able to rank features according with their relevance and to eliminate the least relevant (Saeys et al., 2007). Moreover, other numerous methods to select features have been reported in the literature to improve classifier models, such as feature filtering, and other techniques producing fewer redundant features (Pardoel et al., 2019). Up to now, the best feature set has yet to be clear. Future studies may begin with multiple features and using section algorithms to tune and eliminate features, by which the best set will be produced.

## Limitations and challenges of AI-based gait evaluation in PD

As well known, the PD diagnosis is mainly reliant on clinical features. However, current AI-related studies on gait evaluation have not adopted current diagnosis criteria. Instead, most studies focused on a single modality rather than multimodal approaches. This is not useful for clinical practice, due to PD features cannot be recognized by deep learning models with the same way as by a human neurologist. For instance, deep learning models detect PD from vectorized brain images instead of clinical characteristics, which does not meet the existing diagnosis criteria (Panch et al., 2019). In addition, the mechanisms behind an AI system, called the “black box,” are unclear when it performs a given prediction (Lee et al., 2017; Varghese, 2020). Neurologists are not encouraged to diagnose PD without concrete evidence. More explainable studies for future clinical practice are warranted, thus offering a clinically trusted AI framework.

The medication state of participants (ON and OFF) affects FOG detection and prediction deeply by altering the motor control, physical abilities, and especially the gait patterns. More FOG occurs with smaller shuffling steps in the OFF state than ON state. If A machine-learning model was trained in an optimal medication state of a subject, it would perform worse when the medication wears off and gait changes. Hence, contextual information about medication state is crucial for PD detection and prediction research.

For training data, its availability and quality may be limited by a difficult recruitment of participants and unpredictability of FOG events. Studies involving machine-learning algorithms may not adequately validate an AI-based FOG detection method with few subjects. On the other hand, datasets cannot be guaranteed to be unbiased even for studies including a large number of participants, since freeze may occur in some specific participants for many times during data collection while no FOG occurs in others. In a detection model proposed by Kwon et al. (2014), only 6 of 20 subjects froze, leading to a person-biased model that over-represent the few participants with FOG data. With the prevalence of machine-learning algorithms, data augmentation techniques and additional tests with more individuals are required for the development of models with unbiased data.

Freezing of gait episodes are visually identified and labeled following data collection as ground truth for detection method validation. Although FOG has been well defined, the criteria for determining the beginning and end of a FOG episode was not identical in different models, which makes between-study comparison problematic (Bachlin et al., 2010; Palmerini et al., 2017; Pardoel et al., 2019). Bachlin et al. (2010) and Mazilu et al. (2013a) have used consistent datasets for input, however, both of them are fewer than 250 FOG episodes. For deep learning, dataset size may be a non-negligible issue.

Feature calculation from AI-based device data is typically performed using data windows. For gait evaluation, window lengths have been reported to range from 0.2 to 32 s (Kwon et al., 2014; Punin et al., 2019). Since the output frequency bin resolution is determined by the number of sample points in the input signal, long windows with more points are desirable for calculation of frequency-based features. Nevertheless, long windows will not permit differentiating within-window short events due to decreased temporal resolution, and may introduce unwanted lags in data classification due to slower process. In general, as found in most studies, 1–4 s windows are preferable (Kwon et al., 2014; Rezvanian and Lockhart, 2016).

Different performance metrics have been used in FOG detection, leading to a more difficult comparison among these methods. For example, a real-time system might emphasize onset detection of freeze, so every data point or window might be classified as FOG or no freeze (Bachlin et al., 2010). Contrarily, a long-term monitoring system evaluates whether the FOG occurrence has been successfully detected, so each freeze episode is regarded as a binary event (Rodríguez-Martín et al., 2017a,b). Experimental procedures and fundamental definitions also varied among studies, such as specificity calculation with features from subjects without FOG or exclusion of FOG events which are shorter than 3 s (Tripoliti et al., 2013; Ahlrichs et al., 2016).

Future studies involving AI-based gait evaluation should detail study population, including sample size, sex, PD severity or stage, the number of FOG episodes and the related duration of each person, as well as medication state. For methods, researchers should clearly state the FOG labeling criterion and basis for calculating performance metrics.

## Conclusion and perspectives

Gait analysis is involved in quantitative evaluation of walking parameters in the study of human movement. In recent times, the development of AI-based techniques has revolutionized the gait evaluation approaches, which was initially conducted just in laboratory conditions and relied on multi-camera motion capture systems with force plates. For example, a variety of wearable sensors with a light weight have allowed clinicians to collect indoor and outdoor moving data conveniently, without being limited by laboratory environments. In addition, the wearable devices have lower costs and power consumption, making them suitable for long-term dynamic monitoring of the gait condition of PD patients. However, this method requires patients to wear corresponding sensor devices on their limbs, which can to some extent impair their daily activities. By contrast, the non-wearable devices can comprehensively access patients information without wearing any equipment. Nonetheless, it is susceptible to external interference such as light and surrounding environment during data collection, and it cannot be tracked and identified for a long term. Therefore, designing novel devices combing the advantages of wearable and non-wearable devices is very necessary in the future. At present, a growing number of AI based applications have been widely used in PD detection, FOG prediction, and gait management to minimize the impact of degenerative condition, relying on their great capability to deal with large datasets and identify intricate patterns in pathological gait. A PD suspects can take a video of their walking event and cycle by using their smartphones for an automatic evaluation of gait, providing valuable information to confirm a diagnosis. Furthermore, AI-based gait-assist systems are potential tools for PD management, which may be beneficial for preventing freezes and decreasing fall-related injury. Although current models efficiency has been steadily increased with more and more complex deep-learning algorithms combined with multiple feature sets, important challenges remain because of the lack of large freezing datasets and highly personalized FOG manifestation, thereby improving gait collection methods and gait analysis methods are urgently. Built upon the already created methods, adopting transfer learning and semi-supervised learning models and adding person-specific elements while preserving model generalization are promising approaches for future research, which will provide an intelligent decision-making system for the auxiliary diagnosis and management of PD patients.

## Author contributions

PW and ZL wrote the first of the manuscript. PW, MW, and BC reviewed and modified the final manuscript. All authors conceived this review, read, critically reviewed, and approved the final manuscript.
